# Editorial: Recent Advances in HBV and HCV Immunology

**DOI:** 10.3389/fimmu.2015.00453

**Published:** 2015-09-04

**Authors:** Lynn B. Dustin, Nirupma Trehanpati

**Affiliations:** ^1^Kennedy Institute of Rheumatology, University of Oxford, Oxford, UK; ^2^Institute of Liver and Biliary Sciences, New Delhi, India

**Keywords:** hepatitis B virus, hepatitis C virus, vaccines, interferons, hepatocellular carcinoma

Viral hepatitis is a major public health problem. While a prophylactic vaccine is now available for hepatitis B virus (HBV), an estimated 240–350 million people worldwide are persistently infected with HBV (1–3). There is not yet an approved vaccine to prevent hepatitis C virus (HCV) infection, and between 130 and 200 million people are believed to be chronically infected worldwide (4–6). Untreated, HBV and HCV can each cause liver inflammation, fibrosis, and cirrhosis, and predispose patients to liver failure and hepatocellular carcinoma (2, 5). These infections are difficult and expensive to treat. Therapy for HBV infection may be lifelong, and a true cure with loss of HBV cccDNA from the liver is rarely achieved (2). IFNα-based therapy was the only option for chronic HCV infection until recently; these regimens were often poorly tolerated and frequently ineffective. An array of direct-acting antiviral drugs has greatly improved the outlook for HCV therapy since 2011 (7), but the expense of the new drugs may limit the number of people who benefit. We still do not understand the immunologic mechanisms leading to chronic HBV and HCV infection, how the immune system controls these viruses, and how the immune system contributes to disease pathogenesis. These topics must be understood in order to develop improved therapies and, at least in the case of HCV, an effective vaccine. Our goal in this Research Topic is to cover advancements toward this understanding.

The immune response in the liver – where HBV and HCV each flourish – is incompletely understood. Many of our insights into the immune responses to HBV and HCV have come from studies of human peripheral blood, with limited comparisons to the cells in the liver. The liver is believed to constitute a tolerogenic environment as it receives portal venous blood rich in microbial products from the gut (8, 9). During persistent infection with either HCV or HBV, virus-specific T cells may decrease in number. The virus-specific T cells that can be found tend to have an exhausted phenotype including expression of inhibitory co-receptors, such as PD-1, CTLA-4, or Tim3 (10).

Age at the time of exposure is an important predictor of the outcome of HBV infection: those infected in the perinatal period or early in childhood are likely to experience chronic infection, while those infected as adults often mount a successful immune response, with elimination of the template cccDNA from the liver (2). Antiviral immune responses wax and wane during chronic HBV infection. In this Topic, Schuch and colleagues review the roles of CD8+ T cells and natural killer cells in HBV infection (11). Sharma and colleagues present a study of Treg cells in HBV-associated hepatocellular carcinoma (12). Rajoriya and colleagues review the roles of γδ T cells in HBV or HCV infection as well as other conditions (13).

HCV causes persistent infection in an estimated 50–85% of those exposed (14). Innate and adaptive immune responses can drive spontaneous clearance of HCV infection. In this Topic, Abdel-Hakeem and Shoukry review the role of the immune response in resolution of HCV infection (15). Sung and colleagues focus on the roles played by CD8+ T cells during acute HCV (16). Genome-wide association studies have highlighted a role for IFNλ in determining the outcome of HCV infection and IFNα-based antiviral therapy; this issue is reviewed by Laidlaw and Dustin (17). Cashman and colleagues discuss the roles of humoral immunity in acute and persistent HCV infection (18). Chronic HCV infection is also associated with extrahepatic disease; Sidharthan and colleagues present gene expression study showing a role for monocytes in HCV-associated mixed cryoglobulinemic vasculitis (19). Because of shared modes of transmission, up to 30% of individuals with HIV infection may also be infected with HCV; co-infected individuals have increased inflammation and accelerated liver damage (14). Cho and colleagues present a study of T cell phenotype, activation, and function in HCV–HIV co-infected patients, highlighting distinct changes in T cell function during co-infection – even when HIV is well controlled with highly active antiretroviral therapy (20).

## Conflict of Interest Statement

The authors declare that the research was conducted in the absence of any commercial or financial relationships that could be construed as a potential conflict of interest.

## Funding

LD is supported by the National Institutes of Health (R01 AI060561 and R01 AI089957).

## References

[1] Ott JJ Stevens GA Groeger J Wiersma ST. Global epidemiology of hepatitis B virus infection: new estimates of age-specific HBsAg seroprevalence and endemicity. Vaccine (2012) 30(12):2212–9. 10.1016/j.vaccine.2011.12.116 22273662

[2] Gish RG Given BD Lai CL Locarnini SA Lau JY Lewis DL Chronic hepatitis B: virology, natural history, current management and a glimpse at future opportunities. Antiviral Res (2015) 121:47–58. 10.1016/j.antiviral.2015.06.008 26092643

[3] Trepo C Chan HL Lok A. Hepatitis B virus infection. Lancet (2014) 384(9959):2053–63. 10.1016/S0140-6736(14)60220-8 24954675

[4] Lavanchy D. Evolving epidemiology of hepatitis C virus. Clin Microbiol Infect (2011) 17(2):107–15. 10.1111/j.1469-0691.2010.03432.x 21091831

[5] Hajarizadeh B Grebely J Dore GJ. Epidemiology and natural history of HCV infection. Nat Rev Gastroenterol Hepatol (2013) 10(9):553–62. 10.1038/nrgastro.2013.107 23817321

[6] Mohd Hanafiah K Groeger J Flaxman AD Wiersma ST. Global epidemiology of hepatitis C virus infection: new estimates of age-specific antibody to HCV seroprevalence. Hepatology (2013) 57(4):1333–42. 10.1002/hep.26141 23172780

[7] Gogela NA Lin MV Wisocky JL Chung RT. Enhancing our understanding of current therapies for hepatitis C virus (HCV). Curr HIV/AIDS Rep (2015) 12(1):68–78. 10.1007/s11904-014-0243-7 25761432 PMC4373591

[8] Thomson AW Knolle PA. Antigen-presenting cell function in the tolerogenic liver environment. Nat Rev Immunol (2010) 10(11):753–66. 10.1038/nri2858 20972472

[9] Racanelli V Rehermann B. The liver as an immunological organ. Hepatology (2006) 43(2 Suppl 1):S54–62. 10.1002/hep.21060 16447271

[10] Rehermann B. Pathogenesis of chronic viral hepatitis: differential roles of T cells and NK cells. Nat Med (2013) 19(7):859–68. 10.1038/nm.3251 23836236 PMC4482132

[11] Schuch A Hoh A Thimme R. The role of natural killer cells and CD8(+) T cells in hepatitis B virus infection. Front Immunol (2014) 5:258. 10.3389/fimmu.2014.00258 24917866 PMC4042360

[12] Sharma S Khosla R David P Rastogi A Vyas A Singh D CD4+CD25+CD127(low) regulatory T cells play predominant anti-tumor suppressive role in hepatitis B virus-associated hepatocellular carcinoma. Front Immunol (2015) 6:49. 10.3389/fimmu.2015.00049 25767469 PMC4341117

[13] Rajoriya N Fergusson JR Leithead JA Klenerman P. Gamma delta T-lymphocytes in hepatitis C and chronic liver disease. Front Immunol (2014) 5:400. 10.3389/fimmu.2014.00400 25206355 PMC4143964

[14] Webster DP Klenerman P Dusheiko GM. Hepatitis C. Lancet (2015) 385(9973):1124–35. 10.1016/S0140-6736(14)62401-6 25687730 PMC4878852

[15] Abdel-Hakeem MS Shoukry NH. Protective immunity against hepatitis C: many shades of gray. Front Immunol (2014) 5:274. 10.3389/fimmu.2014.00274 24982656 PMC4058636

[16] Sung PS Racanelli V Shin EC. CD8(+) T-cell responses in acute hepatitis C virus infection. Front Immunol (2014) 5:266. 10.3389/fimmu.2014.00266 24936203 PMC4047488

[17] Laidlaw SM Dustin LB. Interferon lambda: opportunities, risks, and uncertainties in the fight against HCV. Front Immunol (2014) 5:545. 10.3389/fimmu.2014.00545 25400636 PMC4215632

[18] Cashman SB Marsden BD Dustin LB. The humoral immune response to HCV: understanding is key to vaccine development. Front Immunol (2014) 5:550. 10.3389/fimmu.2014.00550 25426115 PMC4226226

[19] Sidharthan S Kim CW Murphy AA Zhang X Yang J Lempicki RA Hepatitis C-associated mixed cryoglobulinemic vasculitis induces differential gene expression in peripheral mononuclear cells. Front Immunol (2014) 5:248. 10.3389/fimmu.2014.00248 24904592 PMC4034044

[20] Cho H Kikuchi M Li Y Nakamoto N Amorosa VK Valiga ME Induction of multiple immune regulatory pathways with differential impact in HCV/HIV coinfection. Front Immunol (2014) 5:265. 10.3389/fimmu.2014.00265 25071758 PMC4086204

